# Anti-outer Membrane Vesicle Antibodies Increase Antibiotic Sensitivity of Pan-Drug-Resistant *Acinetobacter baumannii*

**DOI:** 10.3389/fmicb.2019.01379

**Published:** 2019-06-18

**Authors:** Weiwei Huang, Qishu Zhang, Weiran Li, Yongjun Chen, Congyan Shu, Qingrong Li, Jingxian Zhou, Chao Ye, Hongmei Bai, Wenjia Sun, Xu Yang, Yanbing Ma

**Affiliations:** ^1^Laboratory of Molecular Immunology, Institute of Medical Biology, Chinese Academy of Medical Sciences and Peking Union Medical College, Kunming, China; ^2^Sichuan Institute for Food and Drug Control, Chengdu, China; ^3^The Second Affiliated Hospital of Kunming Medical University, Kunming, China

**Keywords:** antibiotic resistance, *Acinetobacter baumannii*, antibodies, outer membrane vesicles, outer membrane proteins

## Abstract

*Acinetobacter baumannii* often causes serious nosocomial infections. Because of its serious drug resistance problems, complex drug resistance mechanism, and rapid adaptation to antibiotics, it often shows pan-drug resistance and high fatality rates, which represent great challenges for clinical treatment. Therefore, identifying new ways to overcome antibiotic resistance is particularly important. In this study, mice immunized with *A. baumannii* outer membrane vesicles (AbOMVs) produced high IgG levels for a long time, and this antiserum significantly increased the small molecule intracellular aggregation rate and concentrations. *In vitro* experiments demonstrated that the combined used of anti-AbOMV serum and quinolone antibiotics significantly increased the sensitivity of the bacteria to these antibiotics. Mouse sepsis model experiments demonstrated that delivery of these antibodies using both active and passive immunization strategies significantly improved the susceptibility to quinolone antibiotics, improved the survival rate of mice infected with *A. baumannii*, and reduced the bacterial load in the organs. In a pneumonia model, the combination of serum anti-AbOMVs and levofloxacin improved levofloxacin sensitivity, which significantly reduced the bacterial loads in the lung and spleen compared with those of the antibiotic or antibody alone. This combination also significantly reduced lung inflammatory cell infiltration and inflammatory cytokine aggregation. In this study, the main protein targets that bound to these antibodies were identified. Structural modeling showed that seven of the proteins were porins. Therefore, we speculated that the anti-AbOMV antibodies mainly improved the intracellular aggregation of antibiotics by affecting porins, thus improving susceptibility to quinolone antibiotics. This study provides a method to improve susceptibility to existing antibiotics and a novel idea for the prevention and treatment of pan-drug-resistant *A. baumannii*.

## Introduction

*Acinetobacter baumannii* is widely found in nature and is prone to causing infections in the skin, respiratory tract, and urinary system. It is also an important conditional pathogen in hospitals (An and Su, 2018). At present, the *A. baumannii* infection rate continues to rise with the widespread use of antibacterial drugs and has increased in various invasive procedures, and this bacteria has become the main pathogen responsible for nosocomial infections. Of concern, the degree of antibiotic resistance of *A. baumannii* is extremely severe, and the numbers of multidrug-resistant (MDR) and pan-drug-resistant (PDR) strains in intensive care units in particular are increasing, which not only pose great difficulties for clinical treatment but also represent great challenges for nosocomial infection control (Ben-Chetrit et al., 2018).

The resistance mechanisms of *A. baumannii* include inhibition of membrane permeability, efflux pumps, drug-inactivating enzymes, and drug target changes. When multiple resistance mechanisms work together, *A. baumannii* shows severe drug resistance. Bacteria reduce penetration of antibiotics into the cell by altering the structures or modulating the expression levels of outer membrane proteins (OMPs) to affect their permeability. Additionally, bacteria can initiate efflux systems and prevent antibacterial drugs from reaching their effective therapeutic concentrations in the bacteria, which then can escape the bactericidal effects of the antibiotics (Smani et al., 2014; Krishnamoorthy et al., 2017).

Specific antibodies can activate complement, neutralize toxins and viruses, promote phagocytosis, and function by activating and antagonizing targets (Casadevall and Pirofski, 2004). Currently, antibody drugs have been widely used for infectious and autoimmune diseases and tumor immunotherapy (Pagan et al., 2018; Tang et al., 2018). In immuno compromised patients, lack of antibiotic efficacy is very common, which indicates that clearance of bacterial infections results from a combination of the host immune defense and antibiotic sterilization in the patients. A combination of two fully humanized monoclonal antibodies directed against CDA1 and CDB1 with metronidazole or vancomycin significantly reduced the recurrence of *Clostridioides difficile* infection (Lowy et al., 2010). In addition, antibodies targeting *Pseudomonas aeruginosa* PcrV and Psl effectively increased antibiotic sensitivity (DiGiandomenico et al., 2014). The method, which involves linking antibodies and antibiotics with linker molecules to target intracellular pathogens, is more effective than treatment with antibiotics alone (Mariathasan and Tan, 2017). In addition, the combined use of anti-efflux pump protein SerA antibodies and antibiotics improved susceptibility to antibiotics against *Stenotrophomonas maltophilia* (Al-Hamad et al., 2011). These findings suggest that antibody-antibiotic combination drugs have broad application potential.

The OMPs of *A. baumannii* present an important correlation with bacterial drug resistance. Most OMPs are exposed on the cell surface and thus can easily be bound by antibodies. Therefore, a method that can identify effective antibody-binding OMP targets related to drug resistance and antibodies to reverse bacterial resistance will have great significance. However, studies of regulation of drug resistance *A. baumannii* using antibodies are lacking.

The outer membrane vesicles of *A. baumannii* (AbOMVs), which range in size from 10 to 300 nm, are released and secreted extracellularly from the outer membrane by bacteria during growth. Their natural components are mainly phospholipids, OMPs, lipopolysaccharides (LPSs), and soluble periplasmic proteins (Kulp and Kuehn, 2010). Our previous study showed that immunization with AbOMVs produced high levels of antibodies against *A. baumannii*, which protected mice from infection by a drug-resistant strain (Huang et al., 2014). These anti-AbOMV antibodies work against the major OMPs of *A. baumannii* and activate phagocytes to opsonize and kill the bacteria, but the effects of these antibodies on the function of target proteins have not been reported. In this study, we used AbOMVs to immunize mice and obtained polyclonal antibodies that could increase the aggregation of small molecules in bacterial cells *in vitro* and allow antibiotics to rapidly reach high intracellular concentrations. The results showed that the combined use of the antibodies and quinolone antibiotic could effectively improve antibiotic susceptibility both *in vitro* and *in vivo*. Ten major OMPs that reacted with these antibodies were obtained by mass spectrometry analysis and structure prediction. This study provides a basis for screening of antibody-antibiotic combination drugs that are more effective, target multiple OMPs, and can prevent and treat PDR *A. baumannii* infections.

## Materials and Methods

### Ethics Statement

The animal experimental procedures were approved by the Ethics Committee of Animal Care and Welfare, Institute of Medical Biology, CAMS (Permit Number: SYXK (dian) 2010-0007) in accordance with the animal ethics guidelines of the Chinese National Health and Medical Research Council (NHMRC) and the Office of Laboratory Animal Management of Yunnan Province, China. All efforts were made to minimize animal suffering.

All human participants submitted a signed informed consent form to participate in the study. The protocol complied with the Helsinki Declaration and was approved by the Institutional Review Boards of the Institute of Medical Biology, Chinese Academy of Medical Sciences and Peking Union Medical College.

### Bacterial Strains and Mice

All *A. baumannii* strains were isolated from patients hospitalized at the Second Affiliated Hospitals of Kunming Medical University (Kunming, China). All strains are drug-resistant. The *A. baumannii* ATCC 19606 strain was obtained from the American Type Culture Collection (ATCC). Female C57BL/6N mice (6–8 weeks of age) were maintained under specific pathogen-free (SPF) conditions.

### OMV Preparation

OMV preparation was based on a previously described protocol (Huang et al., 2014). A single Ab112 colony was inoculated and cultured overnight in LB without (named AbOMVs or Ab112-OMVs) or with a sub-minimum inhibitor concentration (sub-MIC) of ceftriaxone (32 μg/mL), amikacin (256 μg/mL), azithromycin (256 μg/mL), ampicillin (256 μg/mL), or levofloxacin (2 μg/mL) under different temperatures (25, 30, and 45°C) or in Mueller-Hinton (MH) medium without antibiotics (named MH-AbOMVs). *A. baumannii* ACTT19606 was cultured in LB medium without antibiotics (named 19606-OMVs). The bacterial culture was centrifuged at 14,000 × *g* for 30 min, and the supernatant was filtered through a 0.45-μm membrane (Millipore, Merck). The filtered fraction was concentrated by ultrafiltration with a 500,000 nominal molecular weight cut off (500,000 NMWC) column (GE Healthcare). The concentrate was ultracentrifuged at 200,000 × *g* for 4 h at 4°C. The vesicle pellets were resuspended in phosphate-buffered saline (PBS; 0.02 mol/L phosphate buffer with 0.15 mol/L NaCl, pH 7.4) and then filtered through a 0.45-μm membrane. The absence of viable bacteria in the OMV preparations was determined by spreading aliquots on agar plates to test for bacterial growth. The OMVs were quantified using the Bradford reagent according to the manufacturer’s instructions.

### Preparation of Outer Membrane Protein Complexes (OMPCs)

Outer Membrane Protein Complex preparation was based on a previously described protocol (McConnell et al., 2011). *A. baumannii* Ab112 was grown in 500 mL of LB medium to an optical density at 600 nm (OD600) of 0.8, and the pelleted bacteria were resuspended in PBS and lysed by sonication. Unlysed cells were removed by centrifugation at 4000 × *g* for 5 min, and the supernatant was centrifuged at 20,000 × *g* for 1 h to pellet the cell envelopes. Inner membranes were selectively solubilized with 5 mL of 2% N-laurylsarcosinate by incubation at 37°C for 30 min. The insoluble fraction was pelleted by centrifugation at 20,000 × *g* for 1 h and then washed with PBS.

### Electron Microscopy

Electron microscopy was based on a previously described protocol (Huang et al., 2014). The OMV sample was fixed with 2.5% cold glutaraldehyde in 0.2 M sodium cacodylate buffer (pH 7.4) for 2 h at 4°C and post-fixed with 1% osmium tetroxide in 0.1 M sodium cacodylate buffer (pH 7.4) for 1 h at 4°C; then, the sample was observed and imaged using a transmission electron microscope (Hitachi) at 80 kV.

### Dynamic Light Scattering (DLS)

Dynamic light scattering was based on a previously described protocol (Huang et al., 2016a). AbOMVs were measured by DLS using the Zetasizer Nano ZS (Malvern Instruments) to detect the size distribution, which was reflected by the polydispersity index (PdI) with a range between 0.0 (monodispersed) and 1.0 (entirely heterodispersed).

### Mouse Immunizations

The mouse immunizations were based on a previously described protocol (Huang et al., 2014). The vaccine was prepared by mixing AbOMVs (isolated from Ab112) with an equal volume of 2 mg/mL of Alum adjuvant (Thermo Scientific). Then, each mouse was immunized subcutaneously three times with 100 μL of the vaccine containing 2 μg or 0.2 μg of AbOMVs per mouse at weeks 0, 2, and 4. An additional group of mice was injected with a mixture of PBS and adjuvant to serve as the control.

### Antibody Measurements Using an Enzyme-Linked Immunosorbent Assay (ELISA)

The ELISA based on a previously described protocol (Huang et al., 2016a). AbOMV-specific IgG responses were measured using an ELISA. Briefly, 96-well plates were coated overnight with 25 μg/100 μL of AbOMVs per well. The plates were incubated with the collected serum samples (diluted 1:1000) incubated with anti-mouse IgG secondary antibodies (diluted 1:10,000, Santa Cruz) and developed with an alkaline phosphatase substrate.

### Assessment of Intracellular Small Molecule Accumulation

This experiment refers to a previously reported research method (Coldham et al., 2010). All strains were cultured overnight at 37°C and used to inoculate fresh medium, which was incubated for an additional 5 h at 37°C. In addition, 10^7^ CFU/100 μL of the strains and 80 μL of anti-AbOMVs or control serum (diluted 1:320) were added to a white 96-well plate and co-incubated for 1 h at 37°C. Hoechst 33258 (HT) (25 μM) was added (20 μL) to each well to a final concentration of 2.5 μM. Fluorescence was read from the top of the wells using excitation and emission filters of 352 and 461 nm, respectively, every 5 min for 15 min on the Synergy4 microplate reader (Biotek) and imaged with a fluorescence microscope (Nikon) at 15 min.

### MICs

The MICs were determined using an agar doubling dilution method similar to that recommended by the Clinical Laboratory Standards Institute (CLSI) (Randall et al., 2001), with the main exception being that Luria-Bertani agar was used instead of Mueller Hinton agar. Bacteria grown overnight at 37°C in LB broth were diluted 1:10 in normal saline and inoculated with a multipoint inoculator onto agar containing suitable dilutions of an antibiotic (ceftriaxone, ciprofloxacin, levofloxacin, amikacin, gentamicin, ampicillin, or imipenem). A preliminary study was performed using bacteria incubated with different serum dilutions (1:1 to 1:1024), and the results suggested that the 1:320 dilution was the best anti-serum dilution. Control or anti-AbOMV serum and bacteria (final concentration of 5 × 10^5^ CFU/mL) was incubated at 37°C for 1 h and then added to the plates. The plates were incubated overnight at 37°C, and the MICs were recorded as the lowest concentration that inhibited growth.

### Kinetic Growth Curve Analyses

Levofloxacin was tested at 1/16 of the MIC (1 μg/mL), 1/4 of the MIC (4 μg/mL) or the MIC (16 μg/mL). Ciprofloxacin was tested at 1/4 of the MIC (8 μg/mL) and combined with anti-AbOMVs or control serum (diluted 1:320). Ab112 at a concentration of 5 × 10^5^ CFU/mL in LB was used for all growth curve assays. Samples were collected at 0, 2, 4, 8, 16, and 24 h, diluted in LB and plated in 10-fold dilutions on LB plates. The plates were incubated at 37°C overnight, and the CFUs were counted.

### Mouse Infection and Antibiotic Therapy

For the active immunization studies using the sepsis model, female C57BL/6N mice were immunized with 2 μg of AbOMVs or the Alum control at weeks 0, 2, and 4. At week 40, ∼1 × 10^7^CFU/200 μL [∼10 × of the median lethal dose (LD50)] of *A. baumannii* Ab112 cells with 10% porcine mucin (w/v; Sigma-Aldrich) were administered intraperitoneally to each mouse. For the passive immunization studies in the sepsis and pneumonia models, the mice were administered the Ab112 strain by intraperitoneal (i.p) or intranasal (i.n) challenge. The bacterial dose of the abdominal cavity was the same as that of the active immunization experiment. The intranasal challenge dose was 50 μL of live Ab112 dissolved in PBS at a concentration of 10^9^ CFU/mL. A total of 50 μL of 30 mg/kg of levofloxacin and 50 μL of anti-AbOMV serum (collected at week 40 from the vaccinated mice) were administered in the tail vein 1 h after bacterial challenge, and the mice were treated every 12 h for 3 days.

### Survival Rates and Bacterial Burdens

This experiment refers to the previous research method (Huang et al., 2016a). For the sepsis and pneumonia model, the mice were monitored continuously for 7 days to determine the survival rate after challenge with Ab112. The bacterial burdens in the lung and spleen were measured 12 h after challenge with *A. baumannii* by plating 10-fold dilutions on LB plates. The plates were incubated at 37°C overnight, and the CFUs were counted. The results are expressed as CFU/g.

### Analysis of Lung Inflammation

The lung inflammation analysis was based on a previously described protocol (Huang et al., 2014). For the pneumonia model, the mice were anesthetized 12 h after infection, and lung was collected. After homogenization, the supernatants were kept for cytokine measurements. The concentrations of cytokines (IL-1β and IL-6) were measured using ELISA (eBioscience) according to the supplier’s instructions. Lung tissues were embedded in paraffin and serially sectioned (5 mm) sagittally. The specimens were stained with hematoxylin and eosin to examine peribronchial and alveolar inflammatory cell accumulation. Lung inflammation was scored according to the following definitions (Noto et al., 2017): 0, no pathology; 1, minimal infiltrates of neutrophils in alveolar spaces; 2, low numbers of neutrophils in alveoli; 3, moderate numbers of neutrophils and hemorrhage in alveoli with occasional lobar involvement and focal necrosis of alveolar-wall neutrophils in bronchioles; 4, marked numbers of neutrophils, consolidation, and widespread alveolar necrosis.

### Immunoblotting and Tandem Mass Spectrometry Analyses

This experiment refers to the previous research method (Huang et al., 2016a). *A. baumannii* Ab112 whole cells (WCs) and OMVs were separated by SDS-PAGE. WCs were transferred to a PVDF membrane. *A. baumannii* Ab112 OMV (AbOMV) antiserum or control serum was used as the primary antibody (diluted 1:1000), and horseradish peroxidase (HRP)-conjugated anti-mouse IgG (Invitrogen) was used as the secondary antibody at a 1:10,000 dilution. The blots were developed with an electrochemiluminescence (ECL) substrate (Thermo Scientific).

Tandem matrix-assisted laser desorption/Ionization (MALDI)-time of flight (TOF)-TOF mass spectrometry analysis was performed by Sangon Biotech (Shanghai) Co., Ltd., to identify the protein bands of interest by SDS-PAGE. The samples were removed directly from the gel.

### Homology Modeling and Sequence Identity Analysis

The homology modeling was based on a previously described protocol (Lin et al., 2013). All proteins were modeled by homology *in silica* using the SWISS-MODEL automated protein structure homology modeling server^1^. The sequence homology of these 10 proteins to approximately 2,832 reported *A. baumannii* strains, and amino acid homology with human proteins or other non-homologous strains that excluded *A. baumannii* was analyzed using NCBI BLAST. The homology was divided into seven intervals (100–96%, 95–91%, 90–86%, 85–81%, 80–76%, 75–71%, and 70–0%); the percentages of sequence homology of the 10 proteins from the entire database are fully displayed in a heat map.

### Statistical Analyses

All statistical analyses were performed using GraphPad Prism 6.0 (GraphPad Software, Inc.). The survival rates were compared using the non-parametric log-rank test. One-way ANOVA with Tukey’s multiple comparison test was used to analyze the IgG levels, cytokine concentrations, histology score, fluorescence units, bacterial growth, and bacterial burden. Differences were considered significant if the *P* value was <0.05. All graphed values represent the mean, and the error bars represent the standard error.

## Results

### Mice Immunized With AbOMVs Were Able to Induce a Prolonged IgG Antibody Response

**FIGURE 1:**
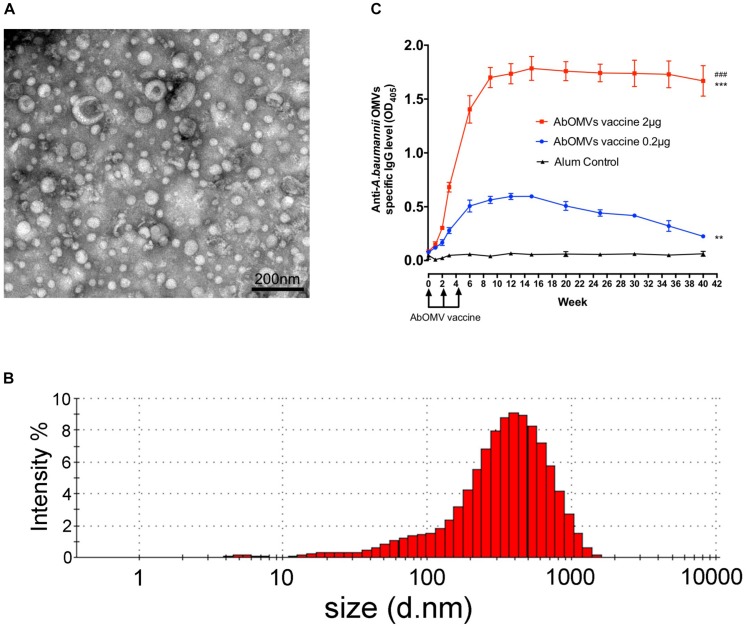
Generation of AbOMVs and mouse immunization. **(A)** OMVs secreted by the Ab112 strain were observed under a transmission electron microscope in a random field of view, bar: 200 nm. **(B)** The OMV diameters were analyzed using a particle size analyzer. **(C)** ELISA was performed to measure the specific anti-AbOMV IgG antibody levels after mice were immunized with the AbOMVs (*n* = 5). ^#^Represents the comparison between the 2 and 0.2 μg dose groups; ^*^represents the comparison between each group and the Alum control group; ^∗∗∗/###^*P* < 0.001; ^∗∗^*P* < 0.01.

The vesicles secreted by PDR *A. baumannii* Ab112 were collected, and the morphology of the AbOMVs were observed under a transmission electron microscope in a random field of view (Figure 1A). Their diameter was determined by a particle size analyzer to be 248.7 ± 5.6 nm, with a polydispersity index ranging from DLS.0.45 ± 0.018 (Figure 1B). C57BL/6N mice were immunized with AbOMVs as a vaccine. After three immunizations, both the 2 and 0.2 μg dose groups produced IgG specific to the AbOMVs compared with the adjuvant control group. The IgG level in the 2 μg dose group was significantly higher than that in the 0.2 μg dose group; the 2 μg dose group sustained a high IgG antibody level for up to 40 weeks, whereas the IgG level in the 0.2 μg dose group had begun to decrease by 15 weeks (Figure 1C). These results showed that the AbOMV vaccine could rapidly induce a high and prolonged IgG response.

### Anti-AbOMV Serum Significantly Increased the Intracellular Aggregation of Small Molecules

**FIGURE 2:**
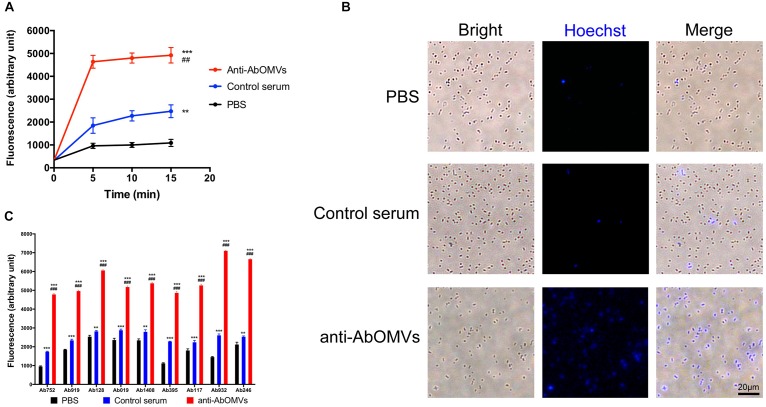
Anti-AbOMV serum significantly increased the aggregation of small molecules in cells. **(A)** The antibodies were incubated with the Ab112 strain for 30 min; then, HT was quickly added, and the dynamic uptake efficiency of HT by the strain was tested every 5 min. The experiment was repeated four times. ^*^Indicates comparison with the PBS group; ^#^indicates comparison with the control serum group. **(B)** After the Ab112 strain was incubated with HT for 15 min, intracellular staining of the bacteria was observed by fluorescence microscopy; the size of each image is the same, bar: 20 μm. **(C)** Fluorescence uptake data were measured 15 min after 9 PDR *A. baumannii* strains were mixed with HT. The experiment was repeated four times. ^∗∗∗^/^###^*P* < 0.001; ^∗∗^/^##^*P* < 0.01.

The uptake rate of the small molecule fluorescent dye HT by drug-resistant *A. baumannii* Ab112 was slow, and HT was enriched intracellularly at a low concentration. The mean fluorescence unit value at 15 min was only 1089. After addition of control serum from normal mice, the mean fluorescence unit value at 15 min was 2475. After addition of the anti-AbOMV serum, the ability of the bacteria to take up HT was rapidly improved, and the mean fluorescence unit value at 15 min was 4922. This experiment demonstrated that addition of anti-AbOMV serum significantly increased HT uptake, suggesting that anti-AbOMV serum could increase the aggregation rate and small molecule concentrations in PDR *A. baumannii* cells (Figure 2A). The intracellular HT aggregation level was observed using fluorescence microscopy. The fluorescence appeared specifically in the bacterial cells, and the number of bacteria that were fluorescently stained after the addition of anti-AbOMV serum was significantly increased compared with that of the serum control and PBS groups (Figure 2B). We used nine clinically isolated PDR *A. baumannii* strains to examine whether this anti-AbOMV serum had universality. The results showed that the anti-AbOMV serum significantly increased the intracellular aggregation of HT in all strains, but the HT uptake efficiency varied among the different strains (Figure 2C).

### Anti-AbOMV Serum Increased the Sensitivity of PDR *A. baumannii* to Antibiotics *in vitro*

**FIGURE 3:**
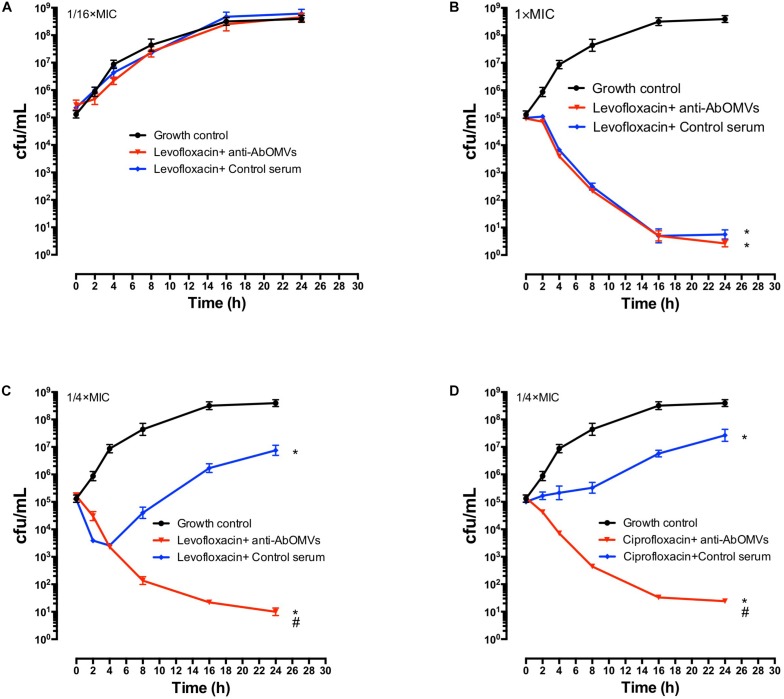
Anti-AbOMV serum improved the sensitivity of PDR *A. baumannii* to antibiotics *in vitro.* Levofloxacin at concentrations of **(A)** 1/16 of the MIC, **(B)** 1x the MIC, and **(C)** 1/4 of the MIC was combined with anti-AbOMV or control serum for 24 h and then viable bacteria (CFU/mL) were counted. **(D)** Ciprofloxacin at a concentration of 1/4 of the MIC was combined with anti-AbOMV or control serum for 24 h and bacteria were counted (CFU/mL). ^*^Indicates anti-AbOMV antiserum or control serum compared with the PBS control; ^#^indicates anti-AbOMVs compared with the control serum group. The experiment was repeated three times. ^*^/^#^*P* < 0.05.

The effect of the anti-AbOMV serum on susceptibility of the Ab112 strain to seven common antibiotics was examined. We found that administration of ceftriaxone together with the anti-AbOMV serum reduced the MIC value of the antibiotic from 256 to 128 μg/mL. Administration of the quinolone antibiotics ciprofloxacin and levofloxacin together with the anti-AbOMV antibodies reduced the MICs of these antibiotics from 32 to 8 μg/mL and from 16 to 4 μg/mL, respectively, which were four times lower than the MICs of the control serum group. The MIC value decreased to 512 μg/mL after the amino acid antibiotic amikacin was combined with the anti-AbOMV antibodies, whereas the MIC of gentamicin still exceeded the highest detection value of 1024 μg/mL. Similarly, the MICs of ampicillin and imipenem combined with the anti-AbOMV serum still exceeded the maximum detection value (Table 1). Combining the anti-AbOMV serum with 1/16 of the levofloxacin MIC could not reverse the antibiotic resistance of the bacteria *in vitro* (Figure 3A). When the MIC concentration of levofloxacin was reached, both the anti-AbOMV and control serum combined with levofloxacin had a bactericidal effect (Figure 3B). When the amount of levofloxacin used was 1/4 of the MIC, combination with the anti-AbOMV serum significantly inhibited bacterial growth; even combination with the control antiserum showed some inhibitory effect, although only a small number of bacteria were killed (Figure 3C). When ciprofloxacin at a concentration of 1/4 of the MIC was used in combination with the anti-AbOMV serum, significant inhibition of bacterial growth was also observed (Figure 3D). These results showed that administration of the anti-AbOMV serum together with quinolone antibiotics could reduce the antibiotic resistance of *A. baumannii*. The reversal of levofloxacin resistance by the anti-AbOMV serum was evaluated in nine different PDR *A. baumannii* strains, and the results showed reduction of drug resistance for 77.78% (7/9) of the strains. Among them, the MIC values were reduced fourfold for five strains and twofold for two strains and did not change for two strains (Table 2). These results showed that the anti-AbOMV antibodies was able to significantly reduce the drug resistance of *A. baumannii in vitro*, especially to quinolone antibiotics.

**TABLE 1:** Determination of MIC values for the Ab112 strain when anti-AbOMV serum was used together with different types of antibiotics.

**Antibiotic**	**MIC (μg/ml)**		
	
	**PBS**	**+Control serum**	**+Anti-AbOMVs**
Ceftriaxone	256	256	128
Ciprofloxacin	32	32	8
Levofloxacin	16	16	4
Amikacin	>1024	>1024	512
Gentamicin	>1024	>1024	>1024
Ampicillin	>1024	>1024	>1024
Imipenem	>1024	>1024	>1024

**TABLE 2:** Determination of MIC values of different strains when anti-AbOMV serum was used together with levofloxacin.

**Strains**	**MIC (μg/ml)**		
	
	**+PBS**	**+Control serum**	**+Anti-AbOMVs serum**
Ab752	32	32	16
Ab919	16	16	4
Ab128	4	4	1
Ab019	16	16	16
Ab1408	16	16	4
Ab395	32	32	16
Ab117	16	16	16
Ab932	32	32	8
Ab246	16	16	4
	% of bacteria to increase antibiotic sensitivity	0%	77.78%

### Anti-AbOMVs Increased Susceptibility of Drug-Resistant *A. baumannii* to Levofloxacin in the Mouse Sepsis Model

**FIGURE 4:**
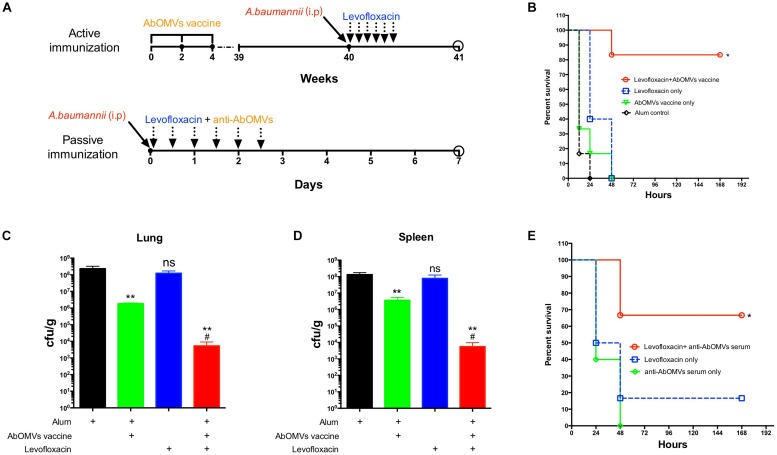
Anti-AbOMV antibodies improved the susceptibility of drug-resistant *A. baumannii* to levofloxacin in the mouse sepsis model. **(A)** Schematic diagram of treatment of *A. baumannii* infection with active and passive immunization combined with antibiotics. **(B)** *A. baumannii* infection was treated with active immunization with AbOMVs combined with levofloxacin, and survival of the mice was observed continuously for 7 days. The bacterial loads of the **(C)** lung and **(D)** spleen 12 h after bacterial challenge. ^*^Represents the comparison between each group and the Alum control group; ^#^represents the comparison with the AbOMV vaccine alone group. **(E)** Survival rate of mice infected with *A. baumannii* and treated with anti-AbOMV serum combined with levofloxacin. ^*^Indicates antibody-antibiotic combination or AbOMV vaccine compared with the Alum control; ^#^indicates anti-AbOMVs combined with antibiotics compared with anti-AbOMVs alone; *N* = 5; ^*^/^#^*P* < 0.05; ^∗∗^*P* < 0.01.

To generate the sepsis model, mice were immunized subcutaneously with AbOMVs three times and challenged intraperitoneally with 10^8^ CFU/mouse (10 × LD50) of strain Ab112 at 40 weeks, followed by administration of levofloxacin at a dose of 30 mg/kg 1 h after challenge. The drug was administered once every 12 h, and the treatment was continued for 3 days. The survival of the mice was observed continuously for 7 days (Figure 4A). The survival rate of the mice infected after immunization with the AbOMVs was significantly increased when they were treated with levofloxacin and remained at 83.33% until day 7 (Figure 4B). The lungs and spleens of the mice were harvested 12 h after bacterial challenge to examine the bacterial loads in their organs. The results showed that mice vaccinated with the AbOMV vaccine had a reduced bacterial load in their lungs and spleen; when levofloxacin was used together with the treatment, the bacterial loads in the lungs (Figure 4C) and spleen (Figure 4D) were significantly reduced compared with those of the vaccination alone group. Antisera were collected from actively immunized mice, and passive immunization combined with antibiotics experiments was performed in the mice. The results showed that the combination of anti-AbOMV antiserum with levofloxacin significantly increased the survival rate of the infected mice compared with those of the groups treated with antibiotics and the anti-AbOMV antiserum alone (Figure 4E). The above experiments showed that immunization with both the AbOMV vaccine and anti-AbOMV serum combined with levofloxacin increased the susceptibility of drug-resistant *A. baumannii* to antibiotics.

### Anti-AbOMV Antibodies Combined With Levofloxacin Significantly Reduced Lung Inflammation in the Mouse Pneumonia Model

**FIGURE 5:**
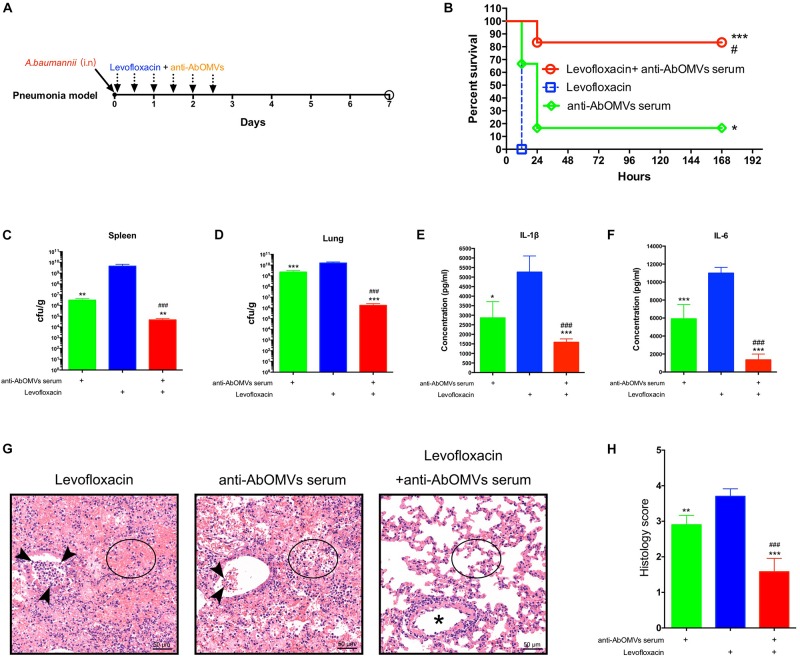
Anti-AbOMV antibodies combined with levofloxacin significantly reduced lung inflammation in mice. **(A)** Schematic diagram of treatment of *A. baumannii* pulmonary infection with passive immunization combined with antibiotics. **(B)** Survival rate of mice infected with *A. baumannii* and treated with anti-AbOMV serum combined with levofloxacin. The bacterial loads in the spleen **(C)** and lung **(D)** were measured 12 h after nasal challenge with Ab112. Approximately 1 mL of lung homogenate supernatant was collected to measure the IL-1β **(E)** and IL-6 **(F)** expression levels by ELISA. **(G)** The lungs were stained with hematoxylin and eosin. Bar, 50 μm. Arrowheads, asterisk, and circles indicate neutrophils, bronchi, and alveolar space, respectively. **(H)** Lung inflammation was scored. ^*^Indicates antibody-antibiotic combination or anti-AbOMVs antiserum compared with the levofloxacin group; ^#^indicates anti-AbOMVs combined with antibiotics compared with anti-AbOMV group; *N* = 6; ^*^P < 0.05; ^∗∗^*P* < 0.01; ^∗∗∗^/^###^*P* < 0.001.

Since *A. baumannii* often causes lung infections in patients, the pneumonia model has greater significance for evaluation of infection. Therefore, after completing the intraperitoneal infection model, we continued to evaluate the ability of the anti-AbOMV antiserum to increase antibiotic sensitivity in the mouse pneumonia model. After intranasal infection with *A. baumannii*, the mice were given anti-AbOMV serum and levofloxacin using a passive immunization approach every 12 h (Figure 5A). The combination of anti-AbOMV antiserum with levofloxacin significantly increased the survival rate of the infected mice compared with those of the groups treated with antibiotics and the anti-AbOMV antiserum alone (Figure 5B). At 12 h post-infection, the anti-AbOMV antiserum combined with levofloxacin significantly reduced the bacterial loads in the mouse spleen (Figure 5C) and lung (Figure 5D) compared to those of the mice administered only antibiotics or the antibodies. The inflammatory cytokines IL-1β (Figure 5E) and IL-6 (Figure 5F) were also significantly lower in the lung homogenate supernatant after anti-serum and antibiotic combination treatment. In addition, lung showed limited tissue damage with mild inflammatory cell infiltration in alveoli after anti-AbOMV serum and antibiotic combination treatment. In contrast, lung of levofloxacin and anti-AbOMV antibodies treatment alone showed more severe neutrophil and lymphocyte aggregation in the peri- and endo-bronchial spaces, and severe hemorrhage and inflammatory cell infiltration in alveoli (Figure 5G). The histology score suggested the tissue destruction and inflammation in the lungs. The results showed that the combination of anti-AbOMV antiserum and levofloxacin significantly reduced the pathology of the lung compared with those obtained in the mice administered antibiotics or antibody treatment alone (Figure 5H). In summary, since the anti-AbOMV antibodies reverse the resistance of *A. baumannii* to levofloxacin, the antibody and levofloxacin combination shows a more effective bactericidal effect and thus effectively reduces inflammation in the lungs.

### The Anti-AbOMV Antibodies Reversed Bacterial Resistance Mainly by Binding to Outer Membrane Porins

**FIGURE 6:**
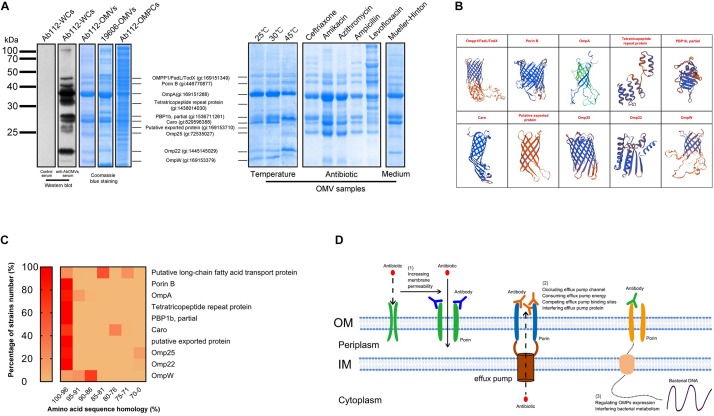
The anti-AbOMV antibodies increased the intracellular aggregation of antibiotics by affecting the porins. **(A)** The immune reaction of anti-AbOMV and control serum with the Ab112 strain was detected by Western blotting; Ab112 OMPCs, 19606-OMVs, and Ab112-OMVs prepared under different culture conditions were detected by SDS-PAGE, and mass spectrometry was used to analyze the corresponding proteins detected by Western blotting. **(B)** The model of the identified proteins was built using the SWISS-MODEL automated protein structure homology-modeling server (http://swissmodel.expasy.org). **(C)** BLAST was used to analyze the amino acid sequence homology of the 10 proteins with approximately 2832 reported *A. baumannii* strains. The heat map shows the homology (displayed in %) of each protein with the proportion (%) of all *A. baumannii* strains. The homology was divided into seven intervals (100–96%, 95–91%, 90–86%, 85–81%, 80–76%, 75–71%, and 70–0%). **(D)** Schematic diagram of the mechanism by which anti-AbOMV antibodies increase the intracellular aggregation of antibiotics.

Western blotting analysis was performed to detect the main bacterial proteins that were immunoreactive with the anti-AbOMV antibodies. The results showed no immune reaction between normal mouse antiserum and *A. baumannii* cells. The anti-AbOMV antiserum had stronger immune reactions with 10 *A. baumannii* proteins. The corresponding AbOMV protein bands were analyzed by mass spectrometry, and these 10 proteins were found to generate a strong immune response (Figure 6A, left). Since these 10 proteins were the major immunogenic proteins, we investigated whether OMPCs or OMVs obtained using different preparation methods would have the same function. To this end, we carefully analyzed the protein compositions of OMVs derived from the standard ATCC19606 strain (19606-OMVs), OMPCs from Ab112 (Ab112-OMPCs), and OMVs derived from the clinical strain Ab112 (Ab112-OMVs) produced under different culture conditions by SDS-PAGE to explore the possibility of using other methods to prepare immunogens and improve antibiotic sensitivity. The results showed that the Ab112-OMPCs contained all of the major antigens, but the composition was more complex. Most of the major proteins on the 19606-OMVs were identical to those on the Ab112-OMVs. The Ab112-OMVs still contained most of the 10 immunogenic proteins after incubation at different temperatures and with different antibiotics and media, but the expression levels of these proteins changed (Figure 6A, right). These results suggested that the bacterial culture conditions had an effect on the protein compositions of OMVs. Therefore, the OMV preparation process must be stable.

Seven proteins had a porin OMP structure based on protein structure homology-modeling (Figure 5B), suggesting that these antibodies enhanced intracellular aggregation of antibiotics mainly by affecting porins. BLAST analysis of the amino acid sequence homology of these 10 proteins demonstrated that they had high sequence similarity in *A. baumannii*. After sequence homology analysis with approximately 2832 *A. baumannii* strains, we found homology with all strains with the following exceptions: 29.61% of the strains for Caro, 24.42% of the strains for the putative long-chain fatty acid transport protein, 13.25% of the strains for Omp25, and 3.79% of the strains for Omp22. The sequence conservation for these proteins in these strains was 81, 76, 70, and 70%, respectively. In most of the other strains, the homology for all 10 proteins was greater than 81%. In fact, most proteins had more than 96% homology among most strains (Figure 6C). Comparison to human proteins showed that OmpA and OmpW had the highest similarity at 34.04 and 32.26%, respectively. No other OMPs had similar proteins.

In summary, this result indicates that the anti-AbOMV antibodies are well conserved among all *A. baumannii* strains and has weak cross-reactivity with human proteins, which is conducive to follow-up studies of antibody drugs.

## Discussion

At present, increasing numbers of multi-drug-resistant bacteria are appearing in the clinic. These bacteria seriously threaten the lives of patients, but development of corresponding antibacterial drugs has progressed slowly. *Enterococcus faecium*, *S. aureus*, *K. pneumoniae*, *A. baumannii*, *P. aeruginosa*, and *E. coli* (ESKAPE) are the most resistant bacteria. To combat these bacterial infections, therapy combining antibodies with antibiotics has been carried out in clinical trials (DiGiandomenico and Sellman, 2015). Therefore, the method of using antibodies to promote antibacterial effects warrants further study (Domenech et al., 2018).

*A. baumannii* outer membrane vesicles can stimulate the body to generate an immune response and long-term immunological memory (Vidakovics et al., 2010; Romeu et al., 2014); therefore, AbOMVs can induce higher IgG levels and a prolonged response as a vaccine (Figure 1C). This study showed that the antibodies could bind to *A. baumannii* OMPs to reduce drug resistance, thereby demonstrating the extensive value of the AbOMV vaccine in preventing infection and reversing drug resistance. We used active immunization experiments with the AbOMV vaccine to demonstrate its ability to induce antibody production and interfere with *A. baumannii* drug resistance (Figure 4B). We also demonstrated that exogenous anti-AbOMV antibodies could increase susceptibility to levofloxacin *in vivo* using passive immunization experiments (Figures 4D, 5). In addition, the AbOMV antibodies reduced the MICs of quinolone antibiotics by fourfold *in vitro* (Figures 3C,D). These results provide a basis for the combined use of antibodies-antibiotics to prevent and treat PDR *A. baumannii* infections. Since our previous study showed that the anti-AbOMV antibodies delivered via both active and passive immunization could inhibit *A. baumannii* infection at the LD50 challenge dose (Huang et al., 2014), the mice in this study were challenged at a dose of 10× the LD50 to highlight the bactericidal effect of the combined use of antibiotics and antibodies *in vivo*. Challenging mice with large doses of *A. baumannii* will kill a large number of immune cells, thereby affecting the bactericidal effect of the AbOMV vaccine when administered alone. However, combined use of antibodies with antibiotics can rapidly kill bacteria, thereby increasing the survival rate of the mice.

Drug-inactivating enzyme-mediated bacterial resistance is very serious and difficult to reverse. For example, oxacillinase, cephalosporinase (AmpC), extended-spectrum β-lactamase (ESBL), metallo-β-lactamase (MBL), glycoside-modifying enzymes, and 16S ribosomal RNA transferase ArmA expression allows *A. baumannii* to acquire resistance to different types of antibiotics (Potron et al., 2015). Bacterial strains with coexistence of these enzymes and other resistance mechanisms exhibit a higher level of resistance. Differences in antibiotic resistance also largely depend on penetration of antibiotics into the cell, and bacteria control the concentrations of intracellular antibiotics through a synergistic relationship between active efflux and outer membrane penetration (Krishnamoorthy et al., 2017). When the external factors affect the OMPs, the membrane permeability is increased or the efflux effect is blocked. Then, the external antibiotic molecules can enter the cell in large quantities, and the antibiotic absorption rate is elevated, which to a certain extent offsets the impact of the efflux effect and the action of dug-inactivating enzymes on antibiotics. Simultaneously, the bacterial intracellular enzymes will leak after the membrane permeability is changed, thereby reducing the concentrations of intracellular drug-inactivating enzymes and enzyme-mediated drug resistance. Similarly, the use of efflux pump inhibitors can effectively reduce drug resistance to quinolone antibiotics (Bhattacharyya et al., 2017). Table 1 shows the effect of the anti-AbOMV antiserum on different types of antibiotics. Our goal was to find the best antibiotic type for combination with the anti-AbOMV antiserum by screening. In this study, the combination of the anti-AbOMV antibodies with quinolone antibiotics (ciprofloxacin and levofloxacin) showed the best combined efficacy. This results may have occurred because resistance of *A. baumannii* to quinolone antibiotics is mainly caused by changes in membrane permeability and effects on active drug efflux. Therefore, a large increase in the intracellular accumulation of antibiotics will have a significant effect on reversing the resistance to these antibiotics. Ampicillin, ceftriaxone, and imipenem are examples of penicillins, cephalosporins and carbapenems, respectively, which are β-lactam antibiotics. *A. baumannii* resistance to β-lactam antibiotics is mainly mediated by β-lactamase and includes a reduction in outer membrane permeability, efflux pump activation and changes in penicillin-binding protein expression. The severe resistance of *A. baumannii* to ampicillin and imipenem may be the result of MDR mechanisms; thus, anti-AbOMV antiserum can increase intracellular accumulation of the drug but does not effectively reverse the resistance of *A. baumannii*. Additionally, the resistance mechanisms of bacteria for aminoglycoside antibiotics (amikacin and gentamicin) include production of aminoglycoside-modifying enzymes (AMEs) and 16S rRNA methylase and decreased outer membrane permeability and drug efflux. Among these mechanisms, AMEs and the 16S rRNA methylase are most important. The AMEs modify the amino and hydroxyl groups on the aminoglycoside antibiotic side chain, and the 16S rRNA methylase methylates the target of the aminoglycoside antibiotic. Both mechanisms can effectively block binding of the aminoglycoside antibiotics to the target, resulting in a loss of antibacterial activity of the antibiotics. The combination of antibiotics and the anti-AbOMV antibodies can increase the intracellular antibiotic concentration, which must counteract the effect of the AMEs and the 16S rRNA methylase (especially ArmA) to prevent antibiotic binding (Liou et al., 2006). If the drug target is highly modified, it may not be able to exert effective bactericidal activity even if a large amount of antibiotics are taken up by bacteria, thus limiting the ability of the anti-AbOMV antiserum to reverse aminoglycoside antibiotic resistance (Doi et al., 2007; Yu et al., 2007). The emergence of strong drug resistance is not the result of a single drug resistance mechanism but often is the result of a combination of MDR mechanisms. These drug resistance mechanisms synergistically cause strong drug resistance in bacteria. Therefore, for strains with strong drug resistance, overcoming only one drug resistance mechanism (i.e., increasing intracellular drug aggregation) will not be sufficient to reverse the drug resistance, which is why the effect of the anti-AbOMV antiserum is limited. In the follow-up study, we will continue to optimize the “antibody-antibiotic” combination strategy and carefully explore the relationship between anti-AbOMV antibodies and antibiotic resistance mechanisms to design more effective “antibody-antibiotic” drugs. Consequently, when designing antimicrobial drugs, consideration of other bacterial resistance mechanisms is purposefully included. At present, prevention and treatment methods for PDR *A. baumannii* include nanoparticle-mediated antibiotic delivery, combined use of antibiotics and antibiotics, combined use of efflux pump inhibitors and antibiotics, and combined use of antimicrobial peptides and antibiotics. However, the combined used of antibodies and antibiotics for the prevention and treatment of *A. baumannii* has not been reported. Therefore, identifying antigen targets that can reverse drug resistance is an important goal.

The bacterial culture temperature, media, and addition of antibiotics can affect the OMV yield and protein composition (Kulp and Kuehn, 2010; McConnell et al., 2011; Yun et al., 2018). The Ab112-OMV preparation method used in this study cultivated Ab112 in LB medium without antibiotics at 37°C. However, whether OMVs collected under other culture conditions would have the same function was not clear. We prepared AbOMVs by changing the bacterial culture conditions (temperature, antibiotics, and media) and examined the differences between OMV protein components before and after these changes. The results showed that some OMPs expression levels on the OMVs were changed in the different preparation (Figure 6A, right). Therefore, we recommend using a standard procedure for OMV preparation. Antibiotic stimulation can increase the OMV yield, which will help improve the preparation efficiency. Therefore, to explore the optimal AbOMV production scheme and more broadly discuss this function of AbOMVs, we cultured Ab112 with five different antibiotics at sub-MIC doses. The harvested OMVs showed some changes in protein bands compared to those of AbOMVs prepared without antibiotics. Although the protein content changed, most proteins were still present on the OMVs. Therefore, we believe that separately analyzing the “antibiotic-OMVs” of interest is necessary to determine whether OMVs prepared under different drug stimulation also have the ability to improve antibiotic susceptibility *in vitro* and *in vivo.*

Differences in OMP expression levels exist between susceptible and resistant *A. baumannii* strains. The membrane proteins associated with antibiotic efflux are often highly expressed in drug-resistant bacteria, but the pore protein associated with antibiotic uptake is often expressed at a low level. Therefore, using AbOMVs secreted by drug-resistant bacteria to prepare antibodies could induce the production of more specific antibodies according to the resistance mechanism of the drug-resistant bacteria. In this study, we used OMVs secreted by PDR bacteria to induce antibodies and reverse bacterial resistance. To explore this function of bacterial OMVs more widely, we also wanted to know whether OMVs from the standard strains had this function. To address this question, we prepared OMVs from the standard strain ATCC19606 (19606-OMVs) and compared the protein components with AbOMVs from clinical strain Ab112. The results showed that the 19606-OMV protein composition was identical to most of the proteins on the AbOMVs (Figure 6A). Therefore, we believe that the 19606-OMVs can perform the same function as the Ab112-OMVs. However, clearly determining whether OMVs from the standard strains have the ability to reverse bacterial resistance also requires more experiments. Furthermore, we were interested in whether OMPCs had the same function as the AbOMVs. Therefore, we extracted OMPCs from the Ab112 strain and compared protein bands with those of the Ab112-OMVs using SDS-PAGE. The results showed that the protein components of the OMPCs were very complex, but the main protein bands in the Ab112-OMVs were all present in the OMPCs (Figure 6A). Therefore, we believe that the OMPCs can induce the body to produce more antibody components, including these 10 major antibodies. However, because the advantages of these 10 proteins may be attenuated by a large number of heteroproteins in the OMPCs, we are not sure of their ability to induce these major antibodies. Regardless, we believe that the protein components of OMVs are simpler than those of OMPCs and that the induced antibodies are more targeted.

In this study, a preliminary analysis of the main protein targets involved in the interaction between the antibodies and bacteria was performed starting with the antibodies targeting the drug-resistant AbOMVs. The 10 main antigen targets we identified were very likely to be the key targets affecting the level of outer membrane permeability or efflux pump activity. The rough structural simulation showed that seven proteins were porins and three were non-porins (Figure 6B). A sequence conservation comparison of these OMPs with known databases demonstrated that these 10 proteins were highly conserved in *A. baumannii* (Figure 6C). Therefore, the anti-AbOMV antibodies generated by this method had extensive cross-reactivity in *A. baumannii*. However, because these proteins exhibit low similarity to human proteins, autoantibody-associated immune disease will not occur during combined use of antibodies and antibiotics. In addition, although our current study was only carried out with *A. baumannii*, we believe that the strategy by which antibodies affect bacterial resistance can be extended to other drug-resistant bacteria, such as drug-resistant *P. aeruginosa*, *K. pneumoniae*, *E. coli* and other ESKAPE pathogens.

The OMPs identified and obtained in this study had a direct association with antibiotic resistance. For *A. baumannii*, the pore-forming activity of the major pore protein OmpA is very low (Iyer et al., 2018). OmpA has been confirmed to be a target of the vaccine against *A. baumannii* (Zhang et al., 2016) and is one of the most important virulence factors of this bacterium (Lee et al., 2010). Furthermore, deletion of the OmpA protein in *A. baumannii* increases susceptibility to antibiotics (Kwon et al., 2017). When the OmpA gene is damaged, the antibiotic susceptibility to chloramphenicol, nalidixic acid, and aztreonam is increased, indicating that OmpA is also involved in drug efflux (Smani et al., 2014). OmpA is both osmotically competent and involved in antibiotic efflux, and therefore the function of OmpA is not singular. These results indicate that OmpA is an important target for the development of new antimicrobial agents and vaccines, but whether anti-OmpA antibodies can reverse bacterial resistance has not been reported. Caro is directly associated with uptake of carbapenem antibiotics and often shows low expression in drug-resistant bacteria (Mussi et al., 2005), but its other functions are not clear. Porin B is an OprB-like protein. The efflux pump BpEAB-OprB system of *Burkholderia* is not associated with resistance to aminoglycosides but can mediate drug resistance to fluoroquinolones, lincosamides, macrolides, and tetracyclines (Mima and Schweizer, 2010). Omp25 is a major porin, and its protein expression level correlates with drug resistance to antibiotics (Rumbo et al., 2013). OmpW is an effective antigen target against *A. baumannii* (Huang et al., 2015). One study reported under-expression of the protein in carbapenem−resistant strains (Tiwari et al., 2012); however, other studies showed that the OmpW expression level in carbapenem-resistant *A. baumannii* was higher than that of the standard strain, indicating that OmpW might also be involved in drug efflux. Therefore, the function of OmpW in antibiotic resistance in *A. baumannii* is not clear. Omp22 may also be an effective vaccine target (Huang et al., 2016b). The 22.5-kDa protein is associated with resistance to antibiotics (Fernandez-Cuenca et al., 2003), but the other functions of this protein are not clear. Taken together, the OMPs identified in this study are associated with drug resistance in *A. baumannii*, and their binding to anti-AbOMV antibodies has the potential to affect their functions and reverse drug resistance.

Efflux pump antibodies in combination with antibiotics improve the sensitivity of drug-resistant bacteria to antibiotics (Al-Hamad et al., 2011). However, when an important OMP is affected by antibodies, bacteria will regulate the expression of other proteins on the outer membrane to form a drug resistance-associated OMP interaction network and enable survival (Wu et al., 2016). Therefore, these proteins work in concert to influence bacterial membrane permeability and efflux, and achieving a good result with a single antibody acting on a single target may be difficult. Anti-AbOMV antibodies are complex antibody components against multiple major porins and have more advantages than a single antibody. However, these anti-AbOMV antibodies also have the possibility of inducing useless antibodies or counteracting antibody components. One of the proteins identified in this study was penicillin-binding protein 1b (PBP1b), which has dual *trans*-transferase and transpeptidase activities and is involved in bacterial peptidoglycan synthesis (Bertsche et al., 2005). PBP1b plays an important role in maintaining cell morphology. Loss of PBP1b impairs bacterial biofilm formation and motility (Kumar et al., 2012; King et al., 2017). Penicillin and ampicillin have high affinity for PBP1b, and binding of these antibiotics interferes with bacterial peptidoglycan synthesis, resulting in bacterial death (Reinert, 2009). The resistance of *A. baumannii* to carbapenem antibiotics is largely due to changes in the PBP protein. When PBP changes, antibacterial drugs do not recognize the bacteria, and the bacteria can successfully escape antibiotic-induced killing (Vila and Marco, 2002; Fernandez-Cuenca et al., 2003; Cayo et al., 2011). In this study, we found that PBP1b was included on the OMVs is a truncated form. This truncated PBP1b was discovered by a genome-wide association study (GWAS) and RNA-seq analysis in December 2018 (gi: 15371121261), but the article has not been published. This protein may represent a cryptic mechanism for drug-resistant *A. baumannii*. We first discovered this truncated PBP1b (aa400-667) on OMVs of drug-resistant *A. baumannii*; the sequence represented the region from amino acids 400 to 667 of the full-length sequence, for a total of 268 amino acids. The protein molecular weight is 28 kDa, which is exactly the same as our SDS-PAGE result. This truncated form of PBP1b is not present in the ATCC 19606 standard strain (Figure 6A), which demonstrates that the truncated protein is unique to drug-resistant strains and may be the product of full-length PBP1b. More interestingly, (1) as the culture temperature increases, the expression level of this truncated PBP1b on OMVs significantly increases. (2) β-Lactam antibiotics, such as ceftriaxone and ampicillin, result in high expression of this truncated PBP1b on OMVs, whereas other types of antibiotics do not affect its expression. This finding may represent an undiscovered, novel and important drug resistance mechanism by which *A. baumannii* escapes β-lactam antibiotics through actively truncating PBP1b under antibiotic pressure. (3) The expression of truncated PBP1b also increases in MH culture medium, which may be related to the ionic strength of different media (Jira et al., 2018). At present, very few studies have investigated the function of PBP1b in *A. baumannii* strains. We cannot clearly explain the function and significance of this truncated PBP1b in bacterial survival, but we believe that this truncated PBP1b is related to bacterial resistance, which warrants in-depth research. In summary, identification of the individual components in anti-AbOMV antibodies has great importance and will aid in the discovery of new bacterial drug resistance mechanisms and the identification of antibody combinations that can effectively reverse bacterial resistance, which is the goal of our future analyses.

*Acinetobacter baumannii* develops drug resistance by reducing the intracellular accumulation of antibiotics, and thus the resistant strain Ab112 shows less HT fluorescence. The control serum was shown to promote HT aggregation, because serum may contain efflux pump inhibitors and complement the effect of bacterial membrane permeability and the efflux pump levels (Ramos-Sevillano et al., 2012; Blanchard et al., 2014). In contrast, the anti-AbOMV serum interacts with porins associated with drug resistance and has been shown to significantly increase intracellular HT aggregation. Although we do not have a good explanation for the bactericidal effect of the combined use of AbOMV antibodies and antibiotics, we believe that they have many properties that can directly or indirectly affect the microorganism, and we speculate that the mechanisms by which anti-AbOMV antibodies promote the intracellular aggregation of antibiotics are as follows (Figure 6D). (1) Anti-AbOMV antibodies affect the stability of the outer membrane and increase its permeability, thereby increasing entry of antibiotics into the cell. (2) A large number of anti-AbOMV antibodies bind to efflux-associated channel proteins, thereby occluding the efflux pump channel, consuming efflux pump energy, competing for the efflux binding sites of antibiotics, or interfering with efflux pump protein components and thus reducing the efflux of antibiotics through this channel (Imamura et al., 2005; Al-Hamad et al., 2011). (3) Anti-AbOMV antibodies regulate the expression levels of bacterial OMPs or interfere with bacterial metabolism (McClelland et al., 2010). Moreover, with the help of antibiotics, vaccines or antibodies can improve AbOMV antibody-mediated opsonophagocytosis and promote the bactericidal effects of active and passive immunity (Ramos-Sevillano et al., 2012).

In summary, the bactericidal effect of the combined use of antibodies and antibiotics is mutually reinforcing and will be a promising technological platform for combating drug-resistant *A. baumannii* infections. In follow-up study, we will continue to study the mechanisms of anti-AbOMV antibodies. First, we will analyze the effects of each individual antibody or antibody combination on bacterial membrane permeability and the efflux pump level, and then RNA-seq will be used to analyze the effects of antibodies on bacterial resistance-related signaling pathways. Thus, the mechanism of anti-AbOMV antibodies in bacterial resistance will be elucidated step by step. We believe that identification of a key target or signaling pathway that regulates bacterial resistance will be of great value for the development of an “antibody-antibiotic” combination drug against PDR *A. baumannii*.

## Conclusion

This study suggested that binding of anti-AbOMV antibodies to the OMPs of drug-resistant *A. baumannii* could increase the intracellular accumulation of antibiotics, thereby reversing the drug resistance of PDR *A. baumannii* and increasing its susceptibility to antibiotics.

## Data Availability

All datasets generated for this study are included in the manuscript.

## Ethics Statement

The animal experimental procedures were approved by the Ethics Committee of Animal Care and Welfare, Institute of Medical Biology, CAMS (Permit Number: SYXK (dian) 2010-0007), in accordance with the animal ethics guidelines of the Chinese National Health and Medical Research Council (NHMRC) and the Office of the Laboratory Animal Management of Yunnan Province, China. All efforts were made to minimize animal suffering. All participants submitted a signed informed consent form to participate in the study. The protocol complied with the Helsinki Declaration and was approved by the Institutional Review Boards of the Institute of Medical Biology, Chinese Academy of Medical Sciences and Peking Union Medical College.

## Author Contributions

WH and QZ designed the topics, experiments, and charts, performed the statistical analysis, and wrote the manuscript. WL and YC assisted with the supplemental experiments and data analysis. CS, QL, and JZ bred the mice, collected the strains, and performed the electron microscopy techniques. CY, HB, WS, and XY provided the experimental support. YM provided the financial support for the study and proposed the amendments.

## Conflict of Interest Statement

The authors declare that the research was conducted in the absence of any commercial or financial relationships that could be construed as a potential conflict of interest.

## Footnotes

1 http://swissmodel.expasy.org
